# Data for the analysis of PolyHIPE scaffolds with tunable mechanical properties for bone tissue engineering

**DOI:** 10.1016/j.dib.2015.09.051

**Published:** 2015-10-23

**Authors:** Robert Owen, Colin Sherborne, Gwendolen C. Reilly, Frederik Claeyssens

**Affiliations:** a Department of Materials Science and Engineering, University of Sheffield, INSIGNEO Institute for in silico medicine, The Pam Liversidge Building, Sir Frederick Mappin Building, Mappin Street, Sheffield S1 3JD, United Kingdom; b Department of Materials Science and Engineering, University of Sheffield, The Kroto Research Institute, North Campus, Broad Lane, Sheffield S3 7HQ, United Kingdom

## Abstract

This article presents data related to the research article titled, ‘Emulsion templated scaffolds with tunable mechanical properties for bone tissue engineering’ (Owen et al., in press) [1]. This data article contains excel files with the results obtained during the mechanical characterisation of 20 acrylate-based PolyHIPE compositions, giving the Young’s modulus, ultimate tensile stress and strain at failure for each specimen tested. Also included are the measurements taken to determine the degree of openness (DOO) of each composition, and the data for the cell viability and alkaline phosphatase (ALP) activity on the emulsion templated scaffolds.

**Specifications Table**

**Table:** 

Subject area	*Biomaterials Science*
More specific subject area	*Bone tissue engineering and materials characterisation*
Type of data	*Tables*
How data was acquired	*Mechanical testing (BOSE ElectroForce* 3200*). SEM images ( Philips XL-*20*). Measurements taken using ImageJ. Fluorescence in vitro assays (FL*_*X*_800 *plate reader, BIO-TEK). Absorbance in vitro assays (EL*_*X*_800 *plate reader, BIO-TEK)*
Data format	*Analyzed*
Experimental factors	*Tensile specimens fabricated from photocured sheets via laser cutting. Scaffolds fabricated using microstereolithography.*
Experimental features	*20 HIPEs with different monomer (*100% *EHA to 100% IBOA,* 25% *intervals) proportions and porosities (*75%*,* 80%*,* 85%*,* 90%*) were prepared by mixing the continuous phase (EHA, IBOA, TMPTA (crosslinker), Hypermer B246-SO-(MV) (surfactant) and (*2,4,6*-trimethylbenzoyl)-phosphine oxide/2-hydroxy-2-methylpropiophenone, 50/50 (photoinitiator)) with the internal phase (water). All 20 compositions were mechanically and physically evaluated. Three compositions with distinctly different stiffness were selected for scaffold manufacture and structured into scaffolds with a multiscale porosity using microstereolithography. Cell viability was determined using resazurin reduction assay and osteogenic differentiation by measuring alkaline phosphatase activity normalised to DNA content.*
Data source location	*Sheffield, United Kingdom*
Data accessibility	*Data is supplied with this article*

**Value of the data**

• The data described here may be helpful to researchers who are performing research where specific mechanical properties are required from a porous polymer, both within tissue engineering and other fields
• A novel method for creating hierarchically porous tissue engineering scaffolds using emulsion templating and microstereolithography
• Air and acrylic acid plasma treatments shown to perform equally with regards to viability and differentiation
• Differentiation ability of a cell line hES-MP 0025 from Cellartis^®^ commercially available but fairly new to the tissue engineering literature

## 1 Data

The data provided here are:

• Young׳s modulus, ultimate tensile stress and strain at failure calculated from the force–displacement curves for each of the 20 PolyHIPE compositions
• Wet mechanical properties for the three compositions selected for cell culture
• Degree of openness values calculated from SEM images using the measurement tool in ImageJ for the physical characterization
• Resazurin reduction cell viability assay fluorescence values
• Normalised ALP activity values

## 2 Experimental design, materials and methods

### 2.1 HIPE synthesis

HIPEs were synthesised with monomer proportions ranging from 100% EHA to 100% IBOA at 25% intervals. The organic component of the continuous phase was formed from the monomers and a crosslinker (TMPTA) at 26.96 wt% of the monomers. A surfactant (Hypermer B246-SO-(MV)) was added at 3 wt% of the organic mass and left to dissolve in a sonic water bath. Finally, a photoinitiator (2,4,6-trimethylbenzoyl)-phosphine oxide/2-hydroxy-2-methylpropiophenone, 50/50) was added at 5 wt% of the organic mass. The internal phase, distilled water, was added at 0.75, 0.80, 0.85 and 0.90 φ to each continuous phase, to produce 20 HIPE compositions. These are referred to by their wt% EHA and nominal porosity. For example, EHA50P85 is a HIPE consisting of 50 wt% EHA and 50 wt% IBOA with a φ of 0.85, and EHA0P75 is a HIPE formed from 100% IBOA with a φ of 0.75.

### 2.2 Fabrication of PolyHIPE sheets for tensile testing

Sheets of PolyHIPE were fabricated from each composition and laser-cut to size based on ASTM D638-10 [2]. HIPE was pipetted into a silicone mould and cured to form a sheet using an automated UV belt curer (GEW Mini Laboratory, GEW engineering UV), washed in acetone and dried overnight. Samples were cut using a laser cutter (Mini 18 Laser, Epilog Laser) with an intensity of 8%, speed of 70% and a frequency of 2500 Hz. The number of passes required was dependent on the porosity and thickness of the PolyHIPE.

### 2.3 Mechanical characterisation of PolyHIPE tensile samples

Samples were tested on a BOSE ElectroForce 3200 mechanical testing machine using a 450 N load cell, an extension rate of 0.02 mm s, a grip distance of 10 mm, and a maximum extension of 6 mm. Each composition was tested and the Young׳s modulus (*E*), ultimate tensile stress (UTS) and percentage elongation at failure determined. The UTS was calculated as the maximum force applied divided by the sample cross sectional area, and percentage elongation at failure expressed as the extension at failure divided by the original distance between the grips (10 mm for all samples). Young׳s modulus of each sample was determined using the gradient of the linear-elastic region of the force–displacement curve. For all samples, the initial point from which this was measured was at an extension of 0.02 mm, and the final point taken was at yield. Compositions selected for cell culture were also tested after soaking in PBS for 1 h. Data Folder 1 contains all the results used to generate Figure 2 in [1].

### 2.4 Physical characterisation of PolyHIPE tensile samples

Scanning electron microscopy (SEM) was used to examine how changes in composition affected the degree of openness (DOO) of the PolyHIPE [3]. Samples were sputter coated with gold and imaged using a Philips XL-20 SEM with an electron beam with energy of 20 kV. Images at 400× magnification were analysed using the measurement tool in Image J [4]. DOO was calculated using the method outlined in [3]. Data Folder 2 contains all the results used to generate Figure 3 in [1].

### 2.5 Scaffold fabrication

Scaffolds were fabricated onto 13 mm glass coverslips. To functionalise the coverslips so that the polymer adhered, they were treated with piranha solution, washed in distilled water, methanol-dried, and added to a solution of 10 wt% 3-methylacryloxypropyltrimethoxysilane (MAPTMS, Polysciences Inc) in toluene. Before use, coverslips were washed in methanol and dried.

Four layer woodpile scaffolds were fabricated from EHA0P80, EHA50P80 and EHA100P80 PolyHIPEs using a single-photon direct-write microstereolithography setup. A subnanosecond laser emitting a wavelength of 355 nm was used a source. Beam delivery was controlled with a shutter and intensity with a pinhole. The beam was focused through a microscope objective and a high precision stage commanded by a motion controller and software was used to translate the focal spot. The laser was focused just above the coverslip-HIPE interface for the bottom layer and the fibre-HIPE interface for each subsequent layer in order to write the scaffold.

For all compositions, a measured laser power of 1.5 mW on the sample was used. To write the scaffold, a layer of HIPE was pipetted onto a functionalised coverslip, placed onto the stage and the first layer fabricated. Additional HIPE was added after the completion of each layer. Once completed, scaffolds were washed with acetone and dried with a heat gun.

### 2.6 Plasma modification of scaffolds

The continuous phase of the HIPE utilises a hydrophobic monomer to form the emulsion. To promote cell adhesion, spreading and proliferation the surface chemistry of the scaffold was altered via plasma modification using either an air plasma (pcAir) or air plasma followed by plasma deposited acrylic acid (pdAAc). Treatments were applied by placing the scaffolds in a cylindrical plasma chamber wrapped with a coil of wire connected to a 13.56 MHz frequency generator. For pcAir scaffolds, the pressure was adjusted to 1.8×10^−1^ mbar and the power set to 50 W to generate the plasma. Samples were exposed to the plasma for 5 min. For pdAAc scaffolds, samples were kept in the chamber after exposure to air plasma and liquid nitrogen added to the cold trap. Once the pressure dropped to 3.0×10^−3^ mbar, a flask of acrylic acid was attached to the inlet and the pressure adjusted to 3.0×10^−2^ mbar. Acrylic acid plasma was then generated for 10 min using a power of 15 W and a flow rate of 2.40–2.50 sccm^−1^.

### 2.7 Cell culture

hES-MPs, mesenchymal progenitors, were used to assess the suitability of the PolyHIPE scaffolds to support osteogenic precursors. Cells were cultured at 37 °C, 5% CO_2_ in basal media (BM), containing Minimum Essential Alpha Medium, 10% foetal bovine serum, 2 mM L-glutamine, and 100 mg/mL penicillin/streptomycin and in gelatine-coated T75 flasks. BM was supplemented with human fibroblastic growth factor at 4 ng/ml and media was changed every 2–3 days.

Cells were cultured in either osteogenesis induction media (OIM) or supplemented media (SM). OIM is BM supplemented with ascorbic acid (50 µg/mL), beta-glycerolphosphate (5 mM) and dexamethasone (100 nM). SM is the same as OIM but without dexamethasone.

Scaffolds were sterilised by soaking in 70 vol% ethanol for 2 h before being washed three times in sterile PBS. Scaffolds were seeded with 75,000 cells at a density of 1500,000 cells/mL and left for 45 min to attach. 1 mL of BM was then added to each well to submerge the scaffolds and incubated overnight. On day 1, scaffolds were transferred to a 12 well plate and 2 mL of OIM or SM added to selected scaffolds. Media was changed every 2–3 days.

### 2.8 Assessment of the suitability of acrylate-based PolyHIPEs for bone tissue engineering

To assess the suitability of the PolyHIPE scaffolds for cell culture, resazurin reduction (RR) assays were performed. The fluorescence is measured using a microplate reader and is correlated with cell viability [5]. 1 mM Resazurin Sodium Salt in dH_2_O was diluted in BM (10 vol %) to create a RR solution. Media was removed from the samples and replaced with 2 ml of RR solution. Well plates were wrapped in foil and incubated for 4 h at 37 °C. 200 µl of the reduced solution was added to a 96-well plate and measured using a spectrofluorometer at an excitation wavelength of 540 nm and an emission wavelength of 630 nm. Data Folder 3 contains all the results used to generate Figure 5 in [1].

### 2.9 Evaluation of the effects of PolyHIPE composition on alkaline phosphatase activity

ALP activity can be used as an early indicator of osteogenic differentiation [6], [7]. Culture media was removed from the scaffolds, they were washed twice in PBS and 500 µl of cell digestion buffer (10 v/v % cell assay buffer (1.5 M Tris–HCl, 1 mM ZnCl_2_, 1 mM MgCl_2_ in deionised water (diH_2_O), 1% Triton-X100, in diH_2_O) was added to each scaffold and incubated for 30 min. Scaffolds were then removed and the lysates transferred to 1.5 mL tubes, vortexed briefly, then stored overnight at 4 °C. The lysates then underwent a freeze-thaw cycle three times (−80 °C 10 min, 37 °C 15 min), before being vortexed for 15 s per sample. Finally, they were centrifuged at 10,000 rpm for 5 min.

10 µl of the lysate was combined with 190 µl of PNPP Phosphatase Substrate (Thermo Scientific, UK) and added to a 96-well plate, then incubated at 37 °C until a slight colour change from colourless to yellow was observed. Absorbance was then measured using a plate reader at a wavelength of 405 nm every minute for 30 min. ALP activity is expressed as nmol of p-nitrophenol per minute (nmol pNP/min), assuming that one absorbance value equals 22.5 nmol of product. This activity is normalised to the amount of DNA as determined using the assay in §1.10 (nmol pNP/min/FU). Data Folder 4 contains all the results used to generate Figure 7 in [1].

### 2.10 PicoGreen^®^ assay

A Quant-iT^™^ PicoGreen^®^ dsDNA Assay (PG) Kit was used to determine the amount of double stranded DNA (dsDNA) present in the cell lysate, indicating cell number. The fluorescence value correlates to the amount of dsDNA present in the sample [8]. 180 µl of the lysate was mixed in a 1:1 ratio with the PG working solution (1:20 Tris-EDTA (TE) buffer (10 mM Tris–HCl, 1 mM EDTA, pH 7.5), 1:200 PG reagent in dH_2_O) in a 1.5 mL tube. This mixed solution was transferred to an opaque well plate, wrapped in foil and incubated at room temperature for ten minutes. Samples were then read using a spectrofluorometer at an excitation wavelength of 485 nm and an emission wavelength of 528 nm.
